# Chemical chaperone treatment reduces intracellular accumulation of mutant collagen IV and ameliorates the cellular phenotype of a *COL4A2* mutation that causes haemorrhagic stroke

**DOI:** 10.1093/hmg/ddt418

**Published:** 2013-09-02

**Authors:** Lydia S. Murray, Yinhui Lu, Aislynn Taggart, Nicole Van Regemorter, Catheline Vilain, Marc Abramowicz, Karl E. Kadler, Tom Van Agtmael

**Affiliations:** 1 Institute of Cardiovascular and Medical Sciences, College of Medical, Veterinary and Life Sciences, University of Glasgow, GlasgowG12 8QQ, UK; 2 Wellcome Trust Centre for Cell-Matrix Research, Faculty of Life Sciences, University of Manchester, ManchesterM13 9PT, UK; 3 Department of Medical Genetics, Hopital Erasme – Université Libre de Bruxelles, Brussels, Belgium

## Abstract

Haemorrhagic stroke accounts for ∼20% of stroke cases and porencephaly is a clinical consequence of perinatal cerebral haemorrhaging. Here, we report the identification of a novel dominant *G702D* mutation in the collagen domain of *COL4A2* (collagen IV alpha chain 2) in a family displaying porencephaly with reduced penetrance. COL4A2 is the obligatory protein partner of COL4A1 but in contrast to most *COL4A1* mutations, the *COL4A2* mutation does not lead to eye or kidney disease. Analysis of dermal biopsies from a patient and his unaffected father, who also carries the mutation, revealed that both display basement membrane (BM) defects. Intriguingly, defective collagen IV incorporation into the dermal BM was observed in the patient only and was associated with endoplasmic reticulum (ER) retention of COL4A2 in primary dermal fibroblasts. This intracellular accumulation led to ER stress, unfolded protein response activation, reduced cell proliferation and increased apoptosis. Interestingly, the absence of ER retention of COL4A2 and ER stress in cells from the unaffected father indicate that accumulation and/or clearance of mutant COL4A2 from the ER may be a critical modifier for disease development. Our analysis also revealed that mutant collagen IV is degraded via the proteasome. Importantly, treatment of patient cells with a chemical chaperone decreased intracellular COL4A2 levels, ER stress and apoptosis, demonstrating that reducing intracellular collagen accumulation can ameliorate the cellular phenotype of *COL4A2* mutations. Importantly, these data highlight that manipulation of chaperone levels, intracellular collagen accumulation and ER stress are potential therapeutic options for collagen IV diseases including haemorrhagic stroke.

## INTRODUCTION

Collagen IV is a major component of the basement membrane (BM), an extracellular matrix structure that provides support and compartmentalization to tissues as well as influences cell behaviour. In the vasculature, a BM surrounds vascular smooth muscle cells and separate them from endothelial cells. Vertebrates express six collagen IV polypeptide chains (α1[IV]–α6[IV]) encoded by the genes *COL4A1–COL4A6*. These polypeptide chains contain a central collagen domain, characterized by Gly-X-Y repeats in which every third amino acid is a glycine residue. In the endoplasmic reticulum (ER), three chains interact to form triple helical protomers and the glycine residues of the collagen domain are critical for helix formation (1). Three distinct protomers occur in vertebrates: α1.α1.α2(IV), α3.α4.α5(IV) and α5.α5.α6(IV)(1), with α1.α1.α2(IV) being present in vascular BMs.

Perinatal haemorrhagic stroke can cause porencephaly, a disease characterized by the presence of a fluid filled cerebral cavity that often leads to congenital hemiplegia. Mutations affecting collagen IV alpha chain 1 (COL4A1) lead to haemorrhagic stroke and porencephaly as well as eye and kidney defects in mice and human patients, thus resulting in a multi-organ disease (2). While the penetrance of cerebral haemorrhaging is close to 100%, phenotypic outcome in mice and clinical manifestations in patients are variable and not all haemorrhages lead to clinical symptoms (2). The phenotypic outcome of *COL4A1* mutations appears to be influenced by the amino acid affected and the position of the affected residue within the alpha chain. For example, in humans, *COL4A1* mutations affecting the CB3 integrin-binding domain of α1.α1.α2(IV) lead to an apparent clinical sub-entity called HANAC (hereditary angiopathy with nephropathy, aneurysm and muscle cramps) syndrome (3,4), and in mice, glycine mutations result in more severe phenotypes than mutations affecting lysine residues (5). Moreover, data from mouse models also indicate that genetic modifiers and environmental factors can influence phenotype development (6,7).

The disease mechanisms of mutations affecting the α1.α1. α2(IV) protomer remain unknown, but initial analyses of *COL4A1* mutations have revealed that they are associated with BM defects (for review see 8). However, in mice, the presence of BM abnormalities in unaffected tissues such as oesophagus (5) suggest that other factors may also contribute to and/or be necessary for disease manifestation. Intriguingly, ER stress has been detected in *Col4a1* mouse models (5,6,9) raising the possibility that it may be a contributing factor. ER stress can lead to activation of the unfolded protein response (UPR), which aims to alleviate ER stress by reducing protein synthesis and increasing chaperone levels to aid protein folding (10). While the UPR is a protective response, chronic ER stress and UPR activation can lead to apoptosis (10) and become pathogenic (for review see 11).

Here, we identify a novel *COL4A2* mutation in a pedigree with porencephaly. We find that while both patient and unaffected father display BM defects, the ER-retention of COL4A2 is unique to patient cells. This COL4A2 intracellular accumulation leads to chronic ER stress and increased apoptosis, shedding light on the disease causing mechanism. We identify that mutant collagen IV is degraded via the proteasome and that altered basal levels of autophagy or proteasomal degradation are not the genetic modifier in this pedigree. Importantly, treatment of patient cells with the chemical chaperone 4-phenyl butyric acid (PBA) effectively reduces COL4A2 intracellular accumulation, ER stress and apoptosis. Taken together, these results indicate that the ability of cells to cope with mutant collagen folding and ER-retention may be a critical modifier of collagen IV diseases and potentially represent future therapeutic targets.

## RESULTS

### A *COL4A2* mutation leads to haemorrhagic stroke and porencephaly

**Figure 1.**
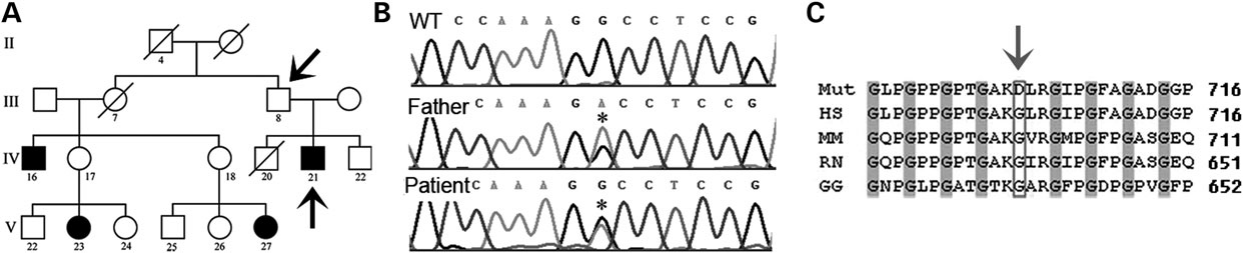
(**A**) A pedigree shows familial dominant porencephaly with reduced penetrance (adapted from Fig. 1 in 13). Individuals analysed are indicated by black arrows and affected individuals are indicated by black symbols. (**B**) Electropherogram of the *COL4A2^G702D^* mutation in the cDNA of patient, unaffected father and WT: control (asterisk indicates mutation). (**C**) Protein alignment of affected highly conserved glycine residue (grey box and arrow) (Mut: patient; HS: human, MM: mouse, RN: rat, GG: chicken). Alignment is based on Ensembl Havana Sequence COL4A2: ENSP00000353654 (arrow indicates the affected amino acid).

Previous identification of *COL4A1* mutations in patients with porencephaly (12) led us to perform candidate gene mutation analysis on a large pedigree presenting with autosomal dominant porencephaly with reduced penetrance (see Fig. 1 in 13 and Fig. 1A). While sequence analysis of patient IV:21, who has a large porencephalic cyst, excluded *COL4A1* mutations, it identified a base pair change in exon 28 of *COL4A2* (Fig. 1B) that is predicted to substitute a glycine residue of a Gly-X-Y repeat for aspartic acid (G702D) (Fig. 1C). Sequence analysis of additional family members confirmed segregation of the mutation with the phenotype and that the mutation was absent in 100 unrelated ethnically matched healthy controls, providing strong evidence that *COL4A2 G702D* represents the causative mutation. The glycine residue is highly conserved (Fig. 1C) and located in the collagen domain of COL4A2, which forms the triple helical domain of the α1.α1.α2(IV) protomer.

Renal and ocular examinations did not reveal any kidney or eye defects characteristic of *COL4A1* disease within this pedigree. Moreover, obligate carriers (Fig. 1A) did not exhibit any signs of subclinical cerebrovascular disease (see Fig. 3 in 13), in contrast to the vascular abnormalities observed using MRI in almost 100% of asymptomatic carriers of *COL4A1* mutations (2). Thus, the reduced penetrance of the cerebral haemorrhaging and absence of extra-vascular defects suggest that *COL4A2* mutations may lead to a milder disease than *COL4A1* mutations.

### *COL4A2^G702D^* leads to BM defects

**Figure 2.**
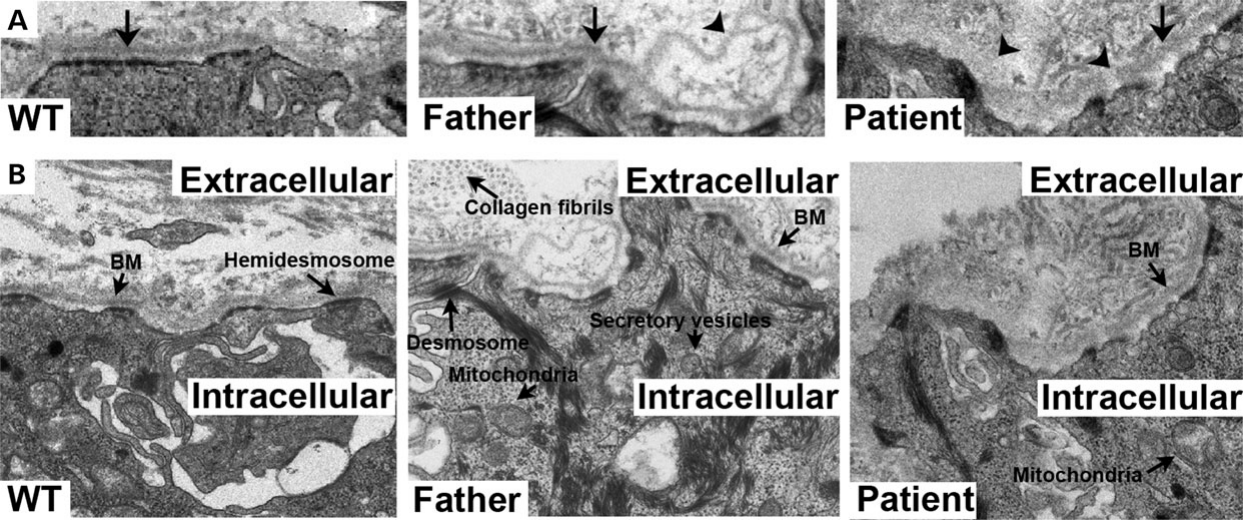
(**A**) EM analysis of unaffected father and patient dermal biopsies show BM defects compared with control (WT) (arrows: intact BM, arrowheads: BM defects). Further annotations are provided in (**B**).

**Figure 3.**
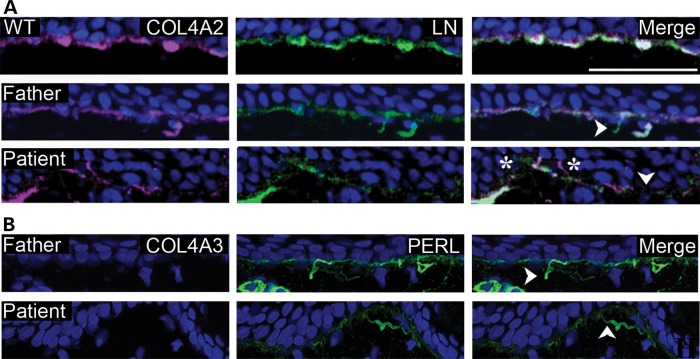
(**A**) Immunofluorescence in dermal biopsies shows focal defects (arrowheads) affecting laminin alpha 1 (LN) (green) and COL4A2 (pink) networks in the patient and unaffected father but not control (WT). Defective COL4A2 deposition into the BM indicated by the absence of co-localization with laminin alpha 1 (star) is seen only in the patient. (**B**) Immunofluorescence analysis against COL4A3 (pink) and perlecan (PERL) (green) identifies that there is no compensation by α3.α4.α5(IV) in unaffected father or patient BM and confirms BM defects (arrow heads) seen in (A). Size bar is 50 µm.

To investigate if defects in the BM contribute to the disease mechanism, dermal biopsies were collected from a patient (IV:21 in Fig. 1A), his unaffected father, who carries the mutation (III:8 in Fig. 1A), and an ethnically matched control. Despite the absence of gross skin defects, electron microscopy (EM) analysis revealed focal BM defects including thinning, duplications and blebbing in the patient and, surprisingly, also in the unaffected father (Fig. 2). Occasional interruptions to the BM were also observed in the patient sample. Immunohistochemical (IHC) analysis against the BM components COL4A2, laminin alpha 1 and perlecan confirmed the presence of these structural BM defects (e.g. interruptions and duplications, Fig. 3A and B). Importantly, in the patient, areas were observed where COL4A2 failed to co-localize with laminin alpha 1, revealing that in this individual the mutation affects the incorporation of COL4A2 into the BM (Fig. 3A). To investigate whether the BM defects lead to ectopic α3.α4.α5(IV) expression, we analysed the expression of COL4A3 in the skin biopsies. No COL4A3 staining was detected indicating that focal absence of α1.α1.α2(IV) does not lead to compensatory α3.α4.α5(IV) expression (Fig. 3B), in contrast to the continued expression of α1.α1.α2(IV) in response to absence of α3.α4.α5(IV) in some Alport syndrome patients (14).

### Increased ER stress is associated with disease development

**Figure 4.**
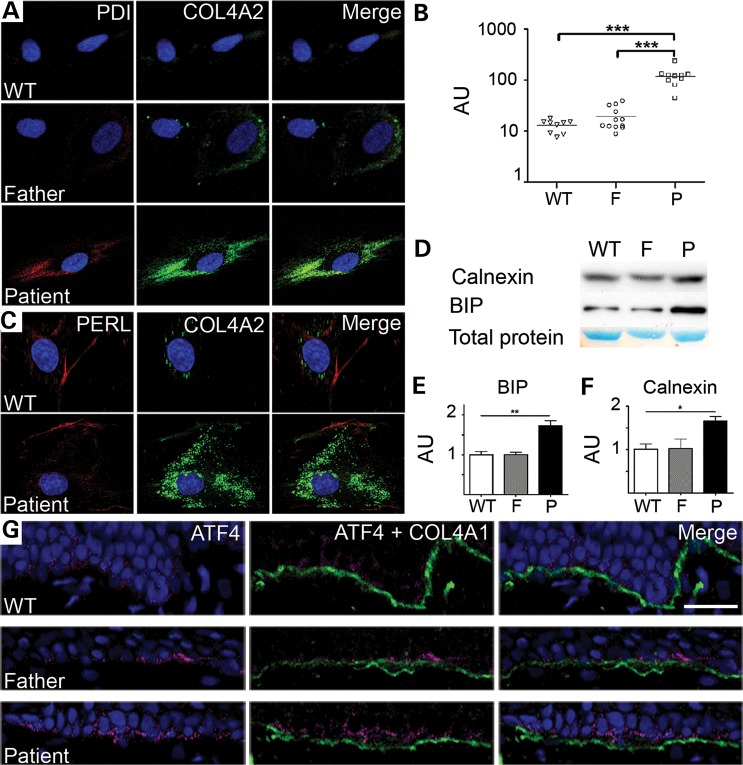
(**A**) Immunofluorescence staining for COL4A2 (green) and PDI (red) in the control (WT), father and patient primary dermal fibroblasts. (**B**) ImageJ analysis of ER volume in patient (P), father (F) and control (WT) cells (WT: 12.8 AU, F: 19.1 AU, P: 118.4 AU) (ImageJ analysis is further demonstrated in Supplementary Material, Fig. S1). (**C**) Immunofluorescence staining for COL4A2 (green) and perlecan (red) in the control (WT) and patient primary dermal fibroblasts. (**D**) Analysis of protein levels of chaperones calnexin, and BIP. Predominant band of total protein stain is given as loading control (entire gel is provided in Supplementary Material, Fig. S3). (**E**) Quantification of BIP protein levels (*n* = 3). (**F**) Quantification of calnexin protein levels (*n* = 3). (**G**) Immunofluorescence staining for COL4A2 (green) and ATF4 (red) in the control (WT), father and patient primary dermal skin biopsy (size bar is 50 µm). Cells were cultured in the presence of ascorbic acid for 72 h prior to analysis (see Materials and Methods). Error bars indicate standard error of measurement. *P < 0.05, **P < 0.01, ***P < 0.001 unpaired *t*-test.

**Figure 5.**
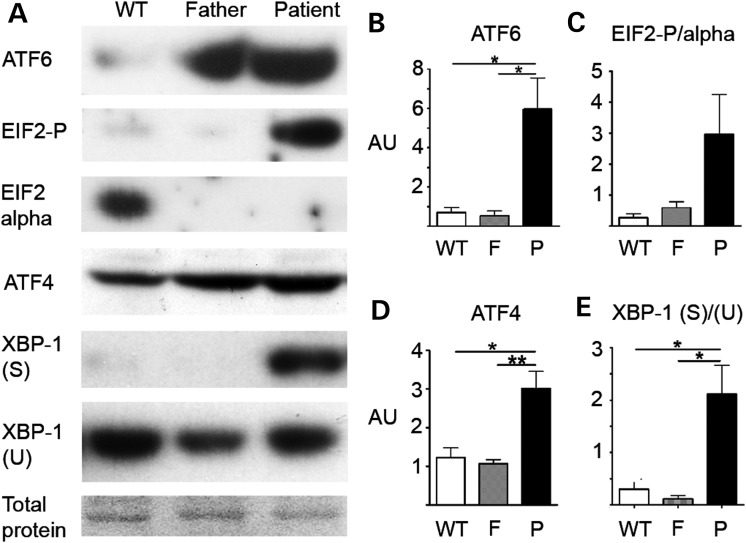
(**A**) Analysis of protein levels of UPR markers activated ATF6 cleavage product, EIF2α, phosphorylated EIF2α (EIF2-P), ATF4 and XBP1 (spliced and un-spliced) in the control (WT), father and patient primary dermal fibroblasts. Predominant band of total protein stain is given as loading control (entire gel is provided in Supplementary Material, Fig. S4). (**B**–**E**) Quantification of ATF6 activated cleavage product, EIF2-P/EIF2 alpha, ATF4 and XBP1 spliced/XBP1 un-spliced, respectively, for control (WT), father (F) and patient (P) cells, using an *n* = 3 for each experiment. Cells were cultured in the presence of ascorbic acid for 72 h prior to analysis (see Materials and Methods for further details). Error bars indicate standard error of measurement. *P < 0.05, **P < 0.01, unpaired *t*-test.

To investigate if defective incorporation of collagen IV into the BM of the patient could be due to a reduction and/or absence of collagen IV secretion, immunocytochemical analysis of COL4A2 localization was performed using primary dermal fibroblasts. This showed increased intracellular staining of COL4A2 and COL4A1 in patient cells compared with the unaffected father and control (Fig. 4A and Supplementary Material, Fig. S1); although both patient and unaffected father express the mutant *COL4A2^G702D^* allele (Fig. 1B). In the patient, intracellular COL4A1 and COL4A2 co-localized with the ER markers protein disulphide isomerase (PDI) (Fig. 4A) and a collagen specific chaperone, heat-shock protein 47 (HSP47) (Supplementary Material, Fig. S1), indicating that α1.α1.α2(IV) protomers were retained within the ER. In contrast, antibody staining against perlecan revealed an absence of retention within the ER, confirming that the patient cells did not have a general protein secretion defect (Fig. 4C). As ER retention of misfolded proteins can lead to increased ER volume, indicative of ER stress (10), we performed ImageJ software (NIH) analysis of Z-stacks of cells stained with the PDI antibody to determine relative ER-volumes (for details, see Material and Methods and Supplementary Material, Fig. S2). This revealed that the patient cells had an ∼6.2-fold and ∼9.3-fold larger ER volume compared with cells from the unaffected father and control, respectively (Fig. 4B). Interestingly, while a small proportion of cells from the unaffected father showed COL4A2 accumulation and larger ER volume than the control, the average ER-volume of the cell population was not significantly increased (Fig. 4B). To investigate whether the intracellular accumulation of collagen IV leads to ER stress and UPR activation, the protein levels of the ER resident proteins calnexin and BIP were assessed. This revealed increased levels of these proteins in the patient compared with carrier and control (Fig. 4C–F). Further investigation of the UPR revealed that expression of mutant COL4A2 leads to increased levels of phosphorylated EIF2α, activated ATF6, spliced XBP1, and ATF4 in the patient compared with the unaffected father and the control (Fig. 5). In line with our data of the relative ER-volume and ER-retention of collagen IV in a proportion of cells of the unaffected father (Fig. 4A and B), slightly elevated expression of some UPR markers (e.g. ATF4) in the unaffected father could be detected on some blots. This probably reflects fluctuations in a small proportion of cells from the unaffected father that accumulate collagen IV.

To investigate whether the increased levels of ER stress and UPR activation in the patient cells reflect the *in vivo* situation, we performed IHC analysis against ATF4 on skin biopsies. In the patient, the majority of cells adjacent to the BM displayed elevated ATF4 levels, indicating UPR activation. However, in the skin biopsy of the unaffected father few cells displayed elevated ATF4 levels compared with the control (Fig. 4G); analogous to the data of the fibroblasts. These data indicate that the *COL4A2* mutation leads to ER stress and UPR activation *in vivo*, and that the *in vitro* primary cellular phenotype reflects the *in vivo* phenotype. We attempted to investigate the EM images for signs of swollen ER vesicles, but due to the limited amount of material available, we were unable to perform this analysis. Comparing the data from the patient and the unaffected father shows that the *COL4A2 G702D* mutation induces high levels of ER retention, ER stress and UPR activation in the patient but not the unaffected father. This suggests that differential handling of mutant protein may influence ER-accumulation, ER stress induction and COL4A2 deposition in the BM, and is associated with disease development.

### Chronic ER stress leads to increased apoptosis in the patient cells

**Figure 6.**
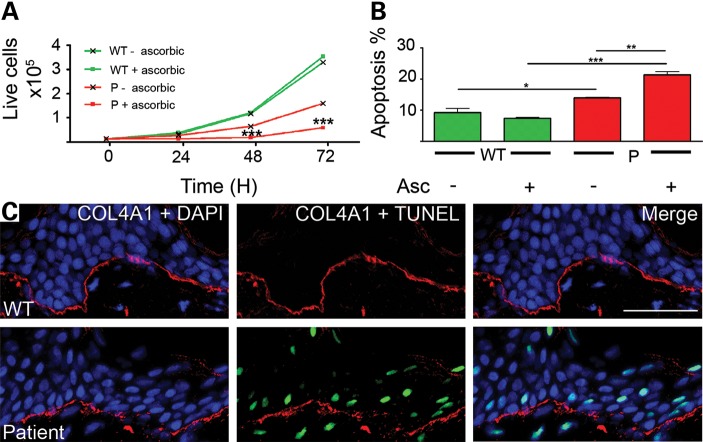
(**A**) Proliferation analysis of patient (P) and control (WT) cells with (+Asc) and without (−Asc) addition of ascorbic acid. (**B**) Percentage of apoptosis positive cells in FACS analysis of control (WT) and patient (P) cells with and without addition of ascorbic acid to media (WT: 9.27%, WT + ascorbic: 7.37%, P: 13.95%, P + ascorbic: 21.33%). (**C**) TUNEL staining (green) assay of control (WT) and patient skin biopsy performed in conjunction with immunofluorescence for COL4A1 (red) and DAPI (blue). Error bars indicate standard error of measurement. *P < 0.05, **P < 0.01, ***P < 0.001 unpaired *t*-test.

While ER stress is a homeostatic response, chronic unabated ER stress can cause apoptosis and be pathogenic (11). To investigate potential cellular consequences of chronic ER stress due to COL4A2 accumulation we measured the proliferation rate of patient and control cells. This revealed an ∼5-fold reduction in patient cells compared with control cells (Fig. 6A). The reduced proliferation was due at least in part to increased levels of apoptosis in the patient (Fig. 6B), which were determined by FACS analysis on cells that were stained for the apoptosis markers Annexin V and propidium iodide; the latter of which also stains necrotic cells . The patient cells exhibited significantly elevated levels of apoptosis as 21.3% of cells underwent apoptosis compared with 7.4% of control cells (Fig. 6B). Interestingly, absence of ascorbic acid in the cell culture medium had no effect on control cells but a 2.5-fold increase in proliferation (Fig. 6A) was observed in patient cells, which was associated with a significant reduction in apoptosis from 21.3 to 13.9% (Fig. 6B). Ascorbic acid is a cofactor for some enzymes required for post-translational modification of collagens and is routinely added to the medium of cultured cells to increase and stabilize expression (15) as well as hydroxylation of all collagens (16). The inclusion/absence of ascorbic acid was not expected to have any significant effect on the control cells which do not carry any collagen mutations. However, the increase in phenotype severity in patient cells in the presence of ascorbic acid strongly indicates that the effects were related to the expression of mutant collagen, presumably collagen IV. To confirm if ER stress also leads to apoptosis in the skin biopsy, terminal deoxynucleotidyl transferase dUTP nick end labelling (TUNEL) was performed which revealed increased apoptosis in the patient compared with the control (Fig. 6C). This strongly suggests that the decreased proliferation and increased apoptosis levels in cells and tissue of the patient are associated with ER retention and, presumably, abnormal folding of collagen IV protein. These data show that the *COL4A2 G702D* mutation which results in ER-retention of misfolded COL4A2 is pathogenic to the cell by causing chronic ER stress, reduced cell proliferation and increased apoptosis.

### Mutant collagen IV is degraded by the proteasome

**Figure 7.**
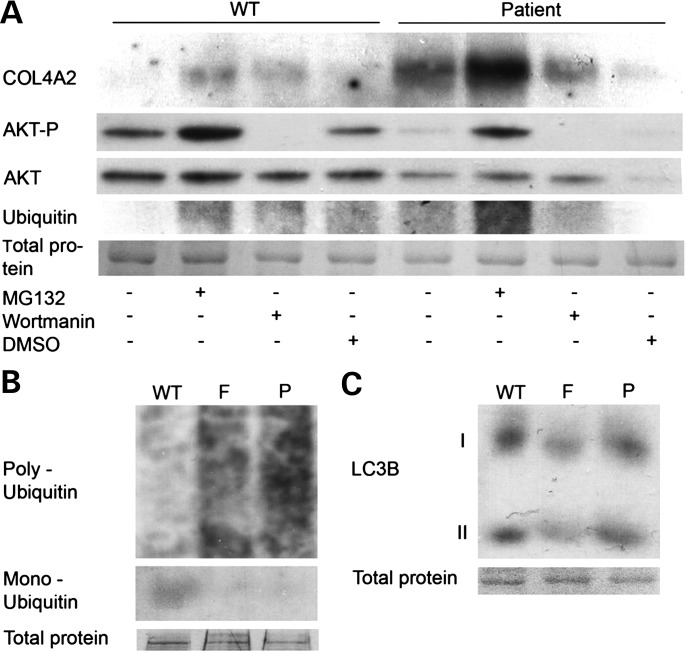
(**A**) Analysis of COL4A2 protein levels by western blot in control (WT) and patient cells. Proteasome and autophagy inhibition by MG132 and wortmanin treatments, respectively. Western blotting against AKT (phosphorylated and pan) and ubiquitin was used as a positive control for wortmannin and MG132 treatment, respectively. Predominant band of total protein stain is given as loading controls (entire gels are provided in Supplementary Material, Fig. S6B, entire ubiquitin blot is provided in Supplementary Material, Fig. S6A). All cells were grown in ascorbic acid containing media 72 h prior to analysis (see Materials and Methods). (**B** and **C**) Analysis of basal proteasome and autophagy levels by ubiquitin protein levels and LC3BI-III, respectively, by western blot in control (WT), father (F) and patient (P) cells.

Recent evidence has indicated that collagen I mutations can be degraded either via the proteasome or autophagy (17). Thus, we set out to determine by which pathway mutant collagen IV is degraded in cells. Western blot analysis was performed for COL4A2 on patient and control cells treated with proteasome and autophagy inhibitors (MG132 and wortmanin, respectively). Increased ubiquitination was used as positive control for the MG132 treatment and loss of phosphorylation of AKT for the wortmannin treatment. Increased intracellular COL4A2 protein levels in patient cells treated with MG132 (Fig. 7A) but not wortmannin, indicating that mutant collagen IV is degraded via the proteasome. This result was verified by treatment of patient cells with the lysosomal protease inhibitors pepstatin A and E64D, which had no effect on intracellular COL4A2 protein levels (Supplementary Material, Fig. S5). To determine whether increased levels of autophagy or proteasome activation in cells of the unaffected father are associated with the different levels of COL4A2 accumulation, western blot analysis was performed for ubiquitin and LC3B I–II cleavage. Ubiquitin levels appeared increased in both patient and father cells compared with the control (Fig. 7B), but no significant differences could be observed in levels of LC3B I–II conversion (Fig. 7C). As no differences were observed in basal levels of autophagy, and proteasomal degradation was equally elevated in patient and father cells, this suggests that basal protein degradation levels do not underlie the variable amounts of intracellular COL4A2 accumulation.

### Treatment of dermal fibroblasts with a chemical chaperone

**Figure 8.**
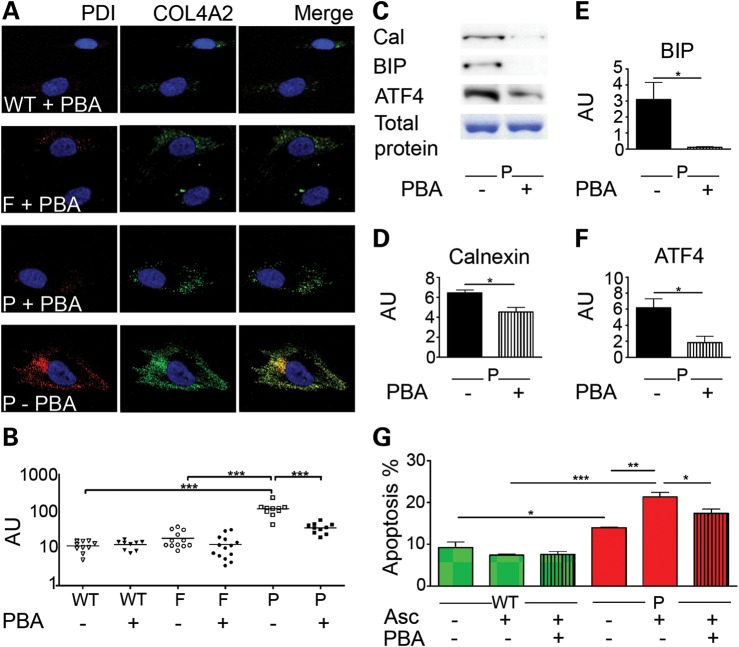
(**A**) Immunofluorescence staining for COL4A2 (green) and PDI (red) in the control (WT), father (F) and patient (P) primary dermal fibroblasts with PBA. (**B**) ImageJ analysis of ER volumes in patient (P), father (F) and control cells (WT) with and without PBA (WT: 12.8 AU, WT + PBA: 11.8 AU, F: 19.1 AU, F + PBA: 13 AU, P: 118.4 AU, P + PBA: 36.3 AU) (confirmation with additional markers shown in Supplementary Material, Fig. S6A and B). (**C**) Analysis of protein levels of chaperones calnexin (cal), BIP and UPR marker ATF4 in the patient's cells following PBA treatment. Predominant band of coomassie gel is given as loading control (entire gel is provided in Supplementary Material, Fig. S7). (**D**–**F**) Quantifications of calnexin, BIP and ATF4 protein levels represented in (C). (**G**) Percentage of apoptosis positive cells in FACS analysis of control (WT) and patient (P) cells with (+asc) and without (−asc) addition of ascorbic and PBA to media (WT: 9.27%, WT + ascorbic: 7.37%, WT + ascorbic + PBA: 7.55%, P: 13.95%, P + ascorbic: 21.33%, P + ascorbic + PBA: 17.37%). Error bars indicate standard error of measurement. *P < 0.05, **P < 0.01, ***P < 0.001 unpaired *t*-test. All cells in figure treated with ascorbic acid 72 h prior to analysis (see Materials and Methods).

Recent evidence has emerged that FDA approved drugs such as 4-PBA have chemical chaperone activity and can reduce ER stress levels (18). As a result, these have gained much attention as a potential treatment option for several diseases associated with ER stress, for example diabetes (19). To investigate if ER stress induced by *COL4A2^G702D^* is amenable to chaperone treatment, cultured cells from the patient unaffected father and control were treated with 10 mM PBA for 24 h. In the patient cells treatment reduced intracellular accumulation of COL4A2, leading to a significant ∼3.3-fold decrease in ER volume, while having no significant effect on the unaffected father or control cells (Fig. 8A and B). The reduction in ER volume corresponded with a reduced level of ER stress as indicated by lower protein levels of the ER stress and UPR markers calnexin, BIP and ATF4 (Fig. 8C–F). Importantly, PBA also ameliorated the levels of apoptosis from 21.3 to 17.4% in the patient cells (Fig. 8G). The significant reduction in phenotype severity confirms the efficacy of PBA in reducing chronic ER stress levels through increased chaperone activity, revealing a potential therapeutic avenue.

## DISCUSSION

Haemorrhagic stroke accounts for 10–15% of all stroke cases, and both genetic and environmental risk factors, such as hypertension and cigarette smoking (20), contribute to disease aetiology. The identification of a novel *COL4A2^G702D^* mutation in a family with porencephaly further underpins the importance of collagen IV mutations in cerebrovascular disease (2). The absence of extra-vascular defects and the reduced penetrance of the porencephaly strongly suggest that *COL4A2* mutations result in a disease of reduced severity and/or affect only a subset of organs compared with *COL4A1* mutations. This is supported by data from mouse models and other families (21–23). Moreover, the reduced penetrance implies that in the absence of an extensive family history, familial haemorrhagic stroke caused by *COL4A2* mutations might be mistakenly classified as sporadic. Thus, stroke due to *COL4A2* mutations might likely be more common in the population than currently appreciated.

Differences in clinical outcome between families with *COL4A1/COL4A2* mutations may be due, in part, to particular consequences of individual mutations (3–5). The *G702D* mutation affects a glycine residue separated by six Gly-X-Y repeats from a 24-nucleotide interruption in the Gly-X-Y repeats of COL4A2 (interruption XIII within COL4A2 (24)). These interruptions are predicted to provide required flexibility to the α1.α1.α2(IV) protomer (25), and while sequence conservation in these interruptions is low, their position within the helical domain is highly conserved (26). Interruption XIII contains two cysteine residues, which form a unique intrachain disulphide bridge leading to a loop protruding from the protomer (24,25). Thus, potential effects on loop formation may exacerbate the expected triple helical distortion caused by the *G702D* mutation. These apparent differences in clinical outcome could also be partially attributed to the unequal composition of α1.α1.α2(IV) protomers (8).

To date, the cellular consequences of *COL4A1*/*COL4A2* mutations remain poorly characterized. By analysing skin biopsies and primary dermal fibroblasts from the patient and unaffected father, we have revealed that the *COL4A2 G702D* mutation leads to BM defects in both individuals. The more severe BM defects in the patient, such as disruptions and defective incorporation of collagen IV, may be due to the observed COL4A2 ER accumulation and resultant reduction in protomer secretion. This association of disease development with BM defects is supported by data from *Col4a1* mutations (5,12). However, BM defects are not always sufficient to cause gross defects (5), suggesting that phenotype development can be influenced by environmental and/or genetic modifiers (6,7). Here, we identify the extent to which mutant collagen is accumulated within the ER, chronic ER stress and UPR activation as critical modifiers. However, it is likely that multiple genetic modifiers for collagen IV mutations exist, and that some of these modifiers may be effective only for particular mutations (e.g. missense mutations). Moreover, the concerted action of several modifiers may be required to avoid disease development.

Our analysis has also established that mutant collagen IV containing a glycine mutation is degraded via the proteasome. This was perhaps surprising as mutant collagen I containing glycine mutations lead to aggregate formation and degradation via autophagy and not the proteasome (17). These data indicate that the degradation pathway employed by the cell may be determined by the inherent structural features of the collagen protomer. For example, collagen IV contains interruptions in the Gly-X-Y repeat while fibrillar collagens such as collagen I do not. However, the apparent discrepancy may also be due to a different experimental set-up as the collagen I mutations were investigated using exogenous expression levels in transfected cells (17), compared with the endogenous expression levels in this study. Regardless, a detailed further analysis of mutant collagen IV is required to investigate the biochemical effects of glycine mutations on collagen IV alpha chains such as aggregation.

The intracellular accumulation of mutant COL4A2^G702D^ leads to ER stress and activation of all three UPR branches: ATF6, XBP1 and PERK-EIF2. In keeping with this, ER stress has been observed in *Col4a1* mutant mice (6,9) and overexpression of mutant collagen in transformed cell lines can cause ER stress; at least under condition of serum starvation (22) where EIF2α is phosphorylated. Furthermore, the ER stress in patient cells is correlated with reduced proliferation and increased apoptosis; and analysis of skin biopsies confirmed the induction of ER stress and apoptosis *in vivo*. These data raise the hypothesis that cellular consequences of chronic ER stress could compound the impact of BM defects on certain tissues, resulting in disease development. They also suggest that ER stress is a general feature of *COL4A1/COL4A2* missense mutations, but further investigations at endogenous levels are required to tease apart any potential mutation-specific effects and whether all mutations activate a similar UPR response in patients.

The presence of uninterrupted COL4A2 within the BM and an absence of ER stress in cells of the unaffected carrier, which appear to confirm the importance of these modifiers, could result from several potential mechanisms. While the identity of these modifiers remains unknown, more efficient intracellular degradation of mutant protein, leading to reduced ER-accumulation and ER stress and secretion of WT protein, is one potential mechanism. However, we failed to detect evidence of increased basal protein degradation or autophagy, suggesting that this is not a modifier in this case. In addition, and/or alternatively, increased protein folding within the ER could prevent accumulation and an ER-stress response, leading to mutant protomer secretion and incorporation into the BM. This is consistent with the unaffected carrier displaying BM defects despite substantial collagen deposition, since studies in heterozygous *Col4a1*/*Col4a2* knockout mice have establish that reduced collagen levels *per se* do not result in BM defects (27). Critically, the use of chemical chaperones that are predicted to increase protein folding (19) was effective in patient cells for reducing both ER-stress levels and apoptosis. These data indicate that, at least *in vitro*, these have efficacy in ameliorating the pathophysiological cellular consequences associated with such mutations. However, the mechanism by which PBA ameliorates the *COL4A2^G702D^* cellular phenotype and its efficacy for other mutations remains to be determined. Several possibilities exist and are not mutually exclusive. For example, PBA treatment may enable the cell to fold the mutant protein more efficiently, which may facilitate mutant protein to be secreted and/or increase mutant protein degradation. While these data are encouraging, the efficacy of PBA as a potential therapeutic needs to be tested in other cell types and animal models as it may lead to additional deleterious effects. For example increased secretion of mutant protein may further increase the severity of the BM defects, and off target effects of the treatment may be identified. The analysis of the *COL4A2^G702D^* mutation performed here utilized a combination of skin biopsies and primary dermal fibroblasts. While we were able to confirm the *in vitro* effects of the mutation on the skin biopsy, these effects will now need to be investigated in the vasculature, endothelial cells, and smooth muscle cells. However, the BM defects (12), defective incorporation of collagen IV into the vascular BM, and UPR activation in aorta of mice with Col4a1 mutations (9) strongly support these findings.

In conclusion, these data suggest that ER accumulation, chaperone activity and variable UPR may act as critical modifiers in the development of collagen IV disease. This highlights an urgent need for *in vivo* analysis of the effects of chemical chaperones on cerebrovascular disease in collagen IV animal models to confirm whether such treatments might benefit patients with collagen IV or other BM mutations that lead to intracellular accumulation of mutant protein.

## MATERIALS AND METHODS

### Dermal biopsy collection

This study was approved by the Ethics review board of Glasgow University and The Hopital Erasme Université Libre de Bruxelles. Following written informed consent three dermal biopsies were collected from the upper, inner arm. The control sample was collected from an ethnically and age-matched control.

### Cell culture and treatments

Primary dermal fibroblast cultures were established from patient and unaffected carrier. Ethnically matched control primary dermal fibroblasts were purchased from TCS Cell Works (UK). Fibroblasts were grown in Chang D medium (Metachem) with 1% penicillin/streptomycin, 10% foetal bovine serum. Experiments were performed in DMEM with GLUTAMAX (Invitrogen) with 1% penicillin/streptomycin, 10% fetal bovine serum. Ascorbic acid treatment (0.25 mM) was administered for 72 h (Sigma).

The proteasome was blocked using 2 μM MG132 for 12 h. The lysosome was blocked using 200 nM wortmanin for 12 h or 10 μg/ml pepstatin A and 10 μg/ml E64D for 24 h. 10 mM PBA treatment (PCI Synthesis) was performed for 24 h. Proliferation analysis was performed using a haemocytometer of live cells distinguished by absence of Trypan Blue staining. FACS analysis of 4000 cells was performed using FITC Annexin V Apoptosis Detection Kit I (BD Pharmingen) as per manufacturer's protocol. Data were obtained from three independent experiments per cell line, per treatment, and statistical analysis was performed using unpaired *t*-test.

### Sequencing

*COL4A1* and *COL4A2* exons were amplified using Bio-X-ACT polymerase (Bioline) and sequenced using BigDye (Applied Biosystems). mRNA was extracted using Trizol (Invitrogen) from fibroblast cell pellets, and cDNA synthesis was prepared using Affinityscript cDNA synthesis kit (Agilent) as per manufacturer's, protocol. Primer sequences are available upon request.

### Electron microscopy

Samples were processed and imaged as previously described (28). Briefly, tissue biopsies were fixed in 2.5% glutaraldehyde in 0.1 M phosphate buffer (pH 7.0) for 24 h at 4°C. Post-fixation specimens were washed in 200 mM phosphate buffer plus 0.1% sodium azide and embedded in epoxyresin (EPON812). Ultra-thin sections were taken on a Reichert-Jung Ultracut (Leica Microsystems, UK) ultramicrotome using a diamond knife (Druker International, The Netherlands) and stained with 2% uranyl acetate in 70% ethanol for 20 min. Sections were counter stained in 0.3% lead citrate in 0.1 M NaOH for five minutes. Tissues were analysed using an FEI Tecnai 12 Twin transmission electron microscope and images were captured using a 2k × cooled CCD camera (F214A, Tietz Video and Image Processing Systems, Gauting, Germany).

### Immunohistochemistry

Cryosections were fixed for 10 min in acetone followed by antigen retrieval using 0.1 M HCl/KCl for 10 min. After blocking in PBS containing 10% FCS, sections were incubated with primary antibodies: COL4A2, COL4A1 and COL4A3 ((1/100) from Dr Yoshikazu Sadu), laminin ((1/1000) from Dr Ulrike Mayer), perlecan ((1/1000) from Dr. Takako Sasaki) and ATF4 (1/400, Stressgen) followed by incubation with fluorescently conjugated secondary antibodies for 1 h at room temperature (Jackson Laboratories). TUNEL was performed using *in situ* cell death detection kit, fluorescein (Roche) as per manufacturer's protocol. Images were captured using an LSM 510 confocal microscope (Zeiss) using a fixed exposure time.

### Immunocytochemistry

Primary dermal fibroblasts were grown on cover slips and processed in the same way as described for immunohistochemistry but with the additional use of antibody PDI (1/400, Cell Signalling) and HSP47 (1/400, Enzo Life Sciences). ER volume was quantified using ImageJ (NIH freeware, http://rsb.info.nih.gov/nih-image). Z-stacks of PDI/HSP47 staining were merged into a single image, and a standard threshold was set to eliminate background signal. The ImageJ program quantified the pixels within a defined area containing 4–7 whole cells, then this value was divided by the number of cells counted to get an average pixel number per cell. Groups of 4–7 cells were used because cells lying directly adjacent were often indistinguishable. Statistical analysis was performed using unpaired *t*-test (WT: *n* = 51, WT + PBA: *n* = 58, carrier: *n* = 75, carrier + PBA: *n* = 85, patient: *n* = 66, patient + PBA: *n* = 76) (Graphpad Prism) on a minimum of three independent experiments, per cell line, per treatment.

### Immunoblotting

Western blotting was performed as previously described (9) except for the collagen blots, which used a wet transfer in contrast to a semi-dry. Protein extracts were prepared using RIPA buffer containing EDTA protease (Roche Applied Science) and phosphatase inhibitors (Phostop Roche). Membranes were blocked with 5% milk before incubation with primary antibodies calnexin (1/8000, Cell Signalling Technology), BIP (1/10 ,000, BD Transduction), ATF4 (1/2500 Stressgen), Ubiquitin (1/1000, Santa Cruz), LC3B (1/500, Novus Biologicals), AKT (1/2000, Cell Signalling) and AKT-P (1/2000, Cell Signalling). Membranes were blocked with 5% BSA before incubation with primary antibodies EIF2 alpha (1/5000 Cell Signalling), phosphorylated EIF2 alpha (1/5000 Cell Signalling), ATF6 (1/2000 Imgenex) and XBP1 (1/2000, Abcam). Protein levels were corrected for coomassie staining of total protein gels ran in parallel with the western blot gels, and measured using ImageJ software analysis (9). Statistical analysis (Graphpad Prism) was performed on a minimum of three independent experiments using unpaired *t*-test.

## AUTHORS’ CONTRIBUTIONS

L.S.M., A.T., Y.L. and T.V.A. performed the experiments. L.S.M., K.E.K., C.V., N.V.R., M.A. and T.V.A. performed data analysis and wrote the manuscript.

## SUPPLEMENTARY MATERIAL

Supplementary Material is available at *HMG* online.

## FUNDING

This work was supported by the Medical Research Council (G0601268) and a Research Councils UK Fellowship (EP/E50036611) (to T.V.A.), and by The Wellcome Trust (091840/Z/10/Z) (to K.E.K.). Funding to pay the Open Access publication charges for this article was provided by a Research Councils UK block grant to The University of Glasgow.

## Supplementary Material

Supplementary Data
