# Supplementary material for: Chemical chaperone treatment reduces intracellular accumulation of mutant collagen IV and ameliorates the cellular phenotype of a COL4A2 mutation that causes haemorrhagic stroke
Source: Hum Mol Genet. 2013 Sep 2;23(2):283–92. doi: 10.1093/hmg/ddt418 (PMC3869351; doi:10.1093/hmg/ddt418)
Supplement: Supplementary Data [file supp_23_2_283__index.html]

Chemical chaperone treatment reduces intracellular accumulation of mutant collagen IV and ameliorates the cellular phenotype of a COL4A2 mutation that causes haemorrhagic stroke — Chemical chaperone treatment reduces intracellular accumulation of mutant collagen IV and ameliorates the cellular phenotype of a COL4A2 mutation that causes haemorrhagic stroke — Chemical chaperone treatment reduces intracellular accumulation of mutant collagen IV and ameliorates the cellular phenotype of a COL4A2 mutation that causes haemorrhagic stroke — Supplementary Data 

# Chemical chaperone treatment reduces intracellular accumulation of mutant collagen IV and ameliorates the cellular phenotype of a *COL4A2* mutation that causes haemorrhagic stroke

## Supplementary Data

Supplementary Data

**Files in this Data Supplement:**

- Supplementary Data - Docx file
