# Supplementary material for: Chemical chaperone treatment reduces intracellular accumulation of mutant collagen IV and ameliorates the cellular phenotype of a COL4A2 mutation that causes haemorrhagic stroke
Source: Hum Mol Genet. 2013 Sep 2;23(2):283–92. doi: 10.1093/hmg/ddt418 (PMC3869351; doi:10.1093/hmg/ddt418)
Supplement: Supplementary Data [file supp_ddt418_ddt418supp.docx]

**Supplemental Data
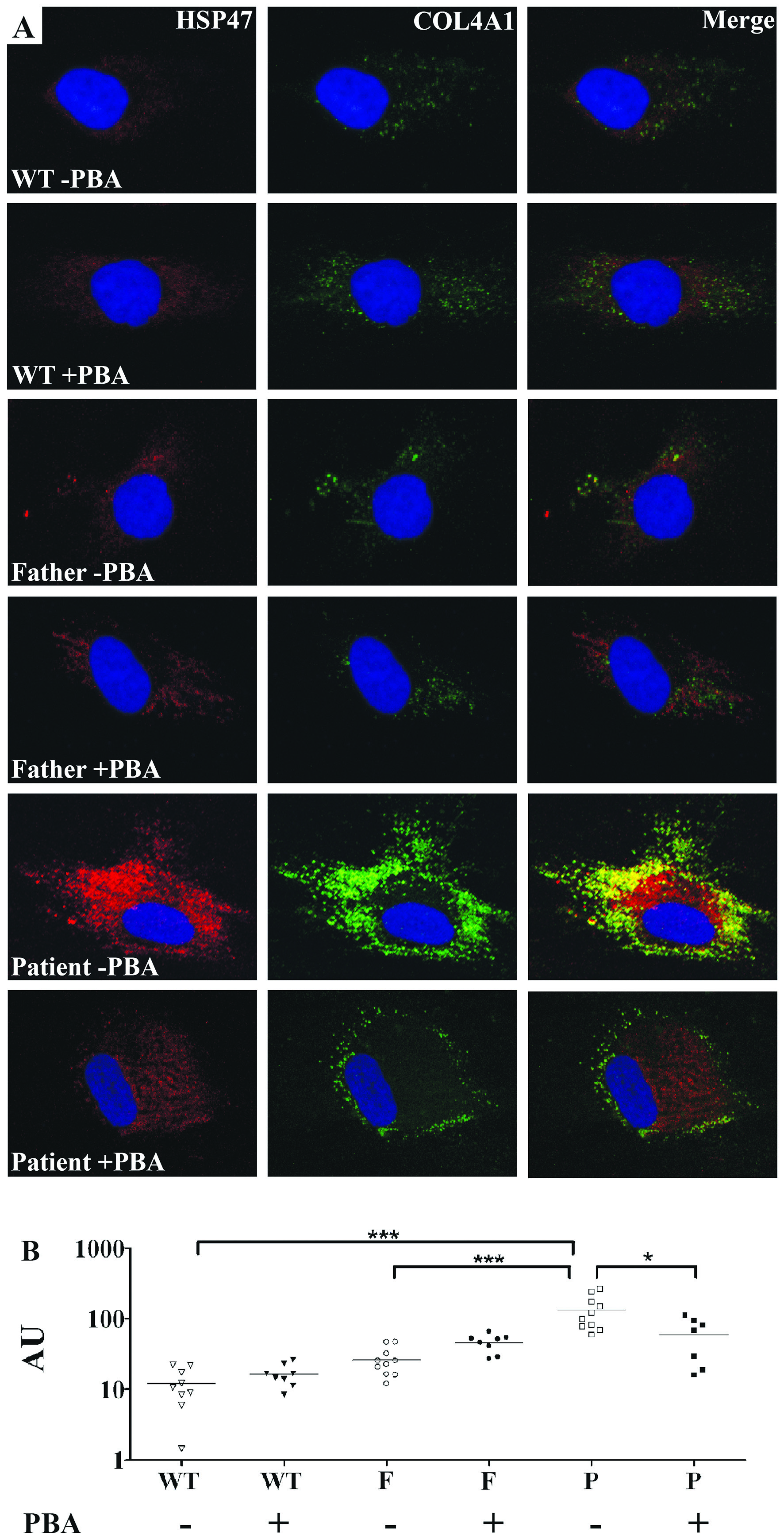
**

**Supplemental Figure 1 (A)** Immunofluorescence staining for COL4A1 (green) and HSP47 (red) in the control (WT), father and patient primary dermal fibroblasts with PBA. (B) Image J analysis of ER volumes in patient (P), father (F) and control cells (WT) with and without PBA (WT: 12.8 arbitrary unit [AU], WT +PBA: 11.8 AU, F: 19.1 AU, F+PBA: 13 AU, P: 118.4 AU, P +PBA: 36.3 AU)


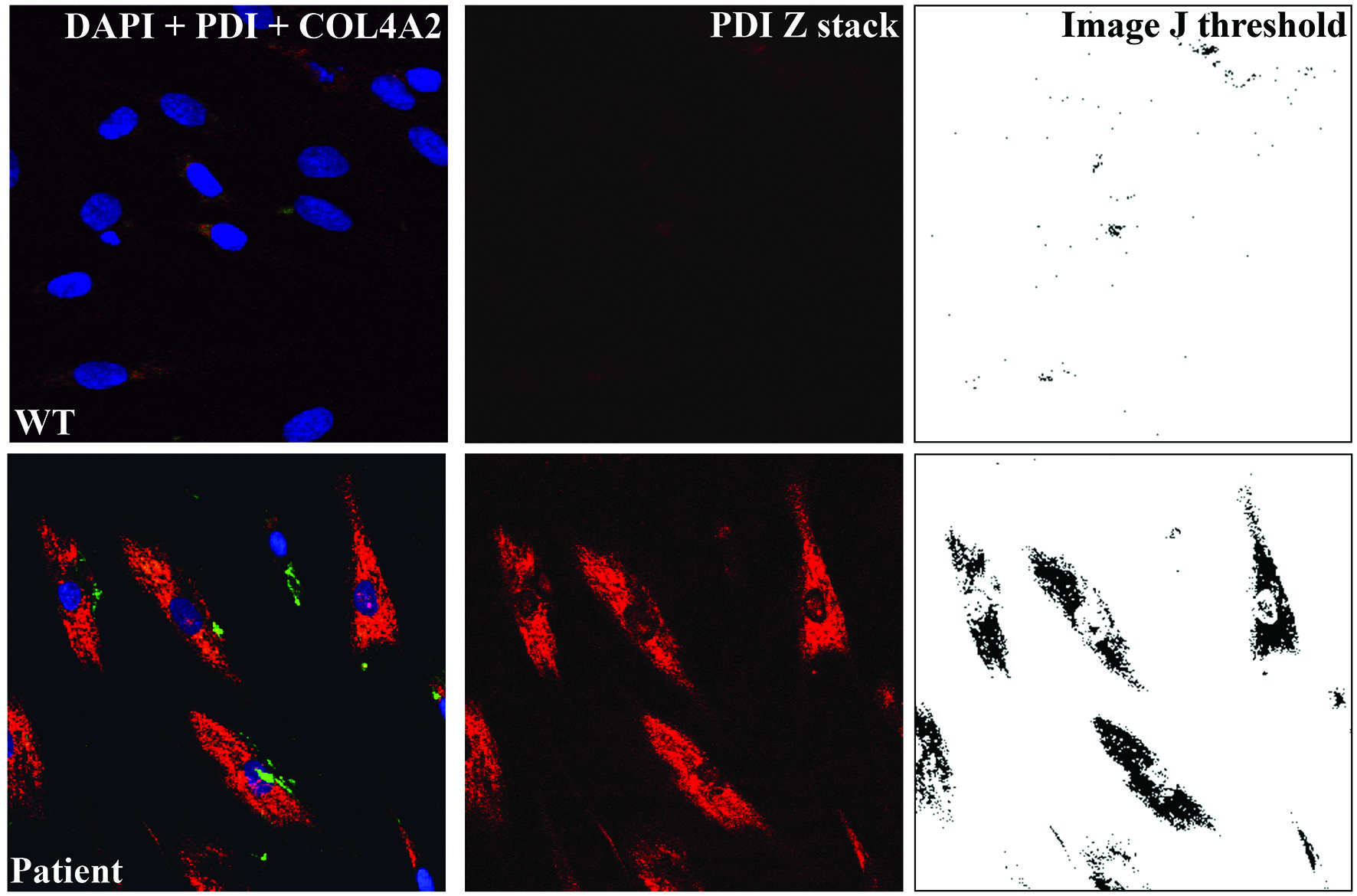


**Supplemental Figure 2:** An overview of the image J ER volume quantification. An example of the immunocytochemistry Z stacked images used to analyse ER volumes and COL4A2 accumulation in Fig 4 (COL4A2 DAPI: blue, PDI: red, COL4A2: green). Image J then selects the PDI staining only and converts the positive staining to black, using a specific threshold (antibody dependent) to distinguish between true staining and background fluorescence. A boundary is drawn so image J measures the pixels from the complete cells only, in the field of vision then this number is divided by the number of cells within the boundary to provide an average. Due to cells often growing directly against each other or at different confluencey, individual cells were not counted. 4-7 cells were grouped and averaged for each measurement and all cells within each field of vision (selected randomly from multiple slides) were included.


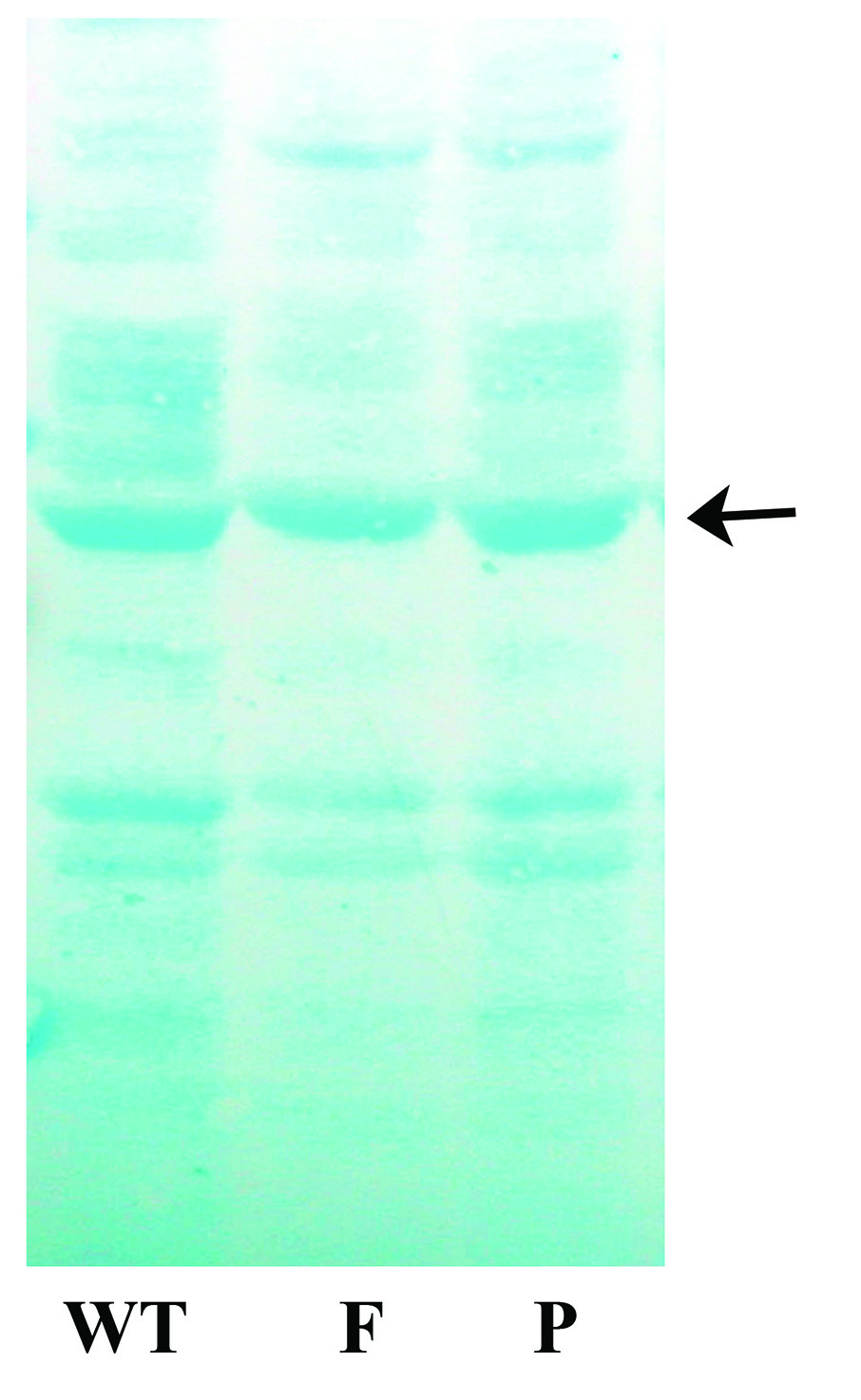


**Supplemental Figure 3:** A reversible total protein stain of the membrane in Fig 4. Protein band used is highlighted by black arrow.


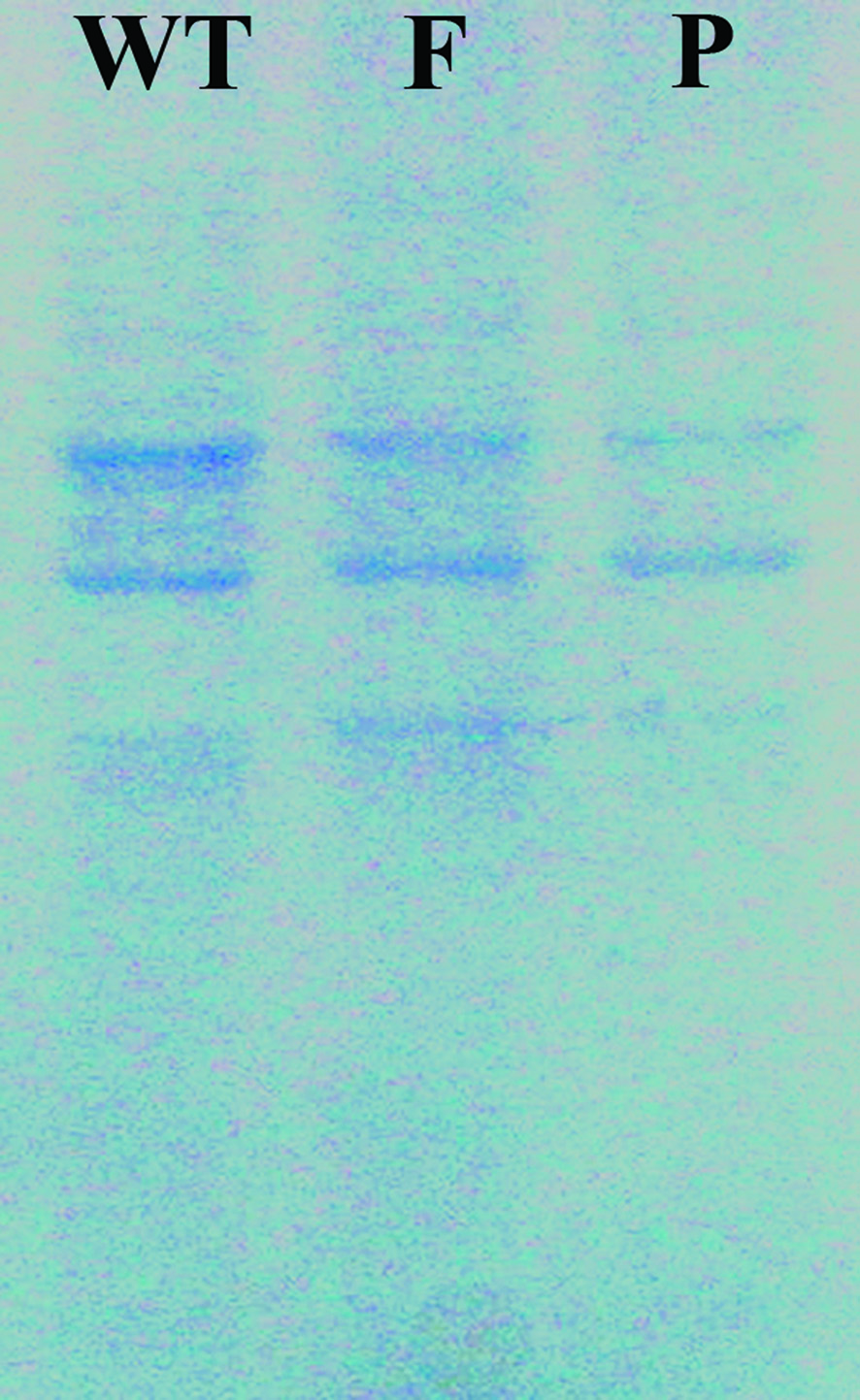


**Supplemental Figure 4:** Entire coomassie stained gel used in Fig 5.

**
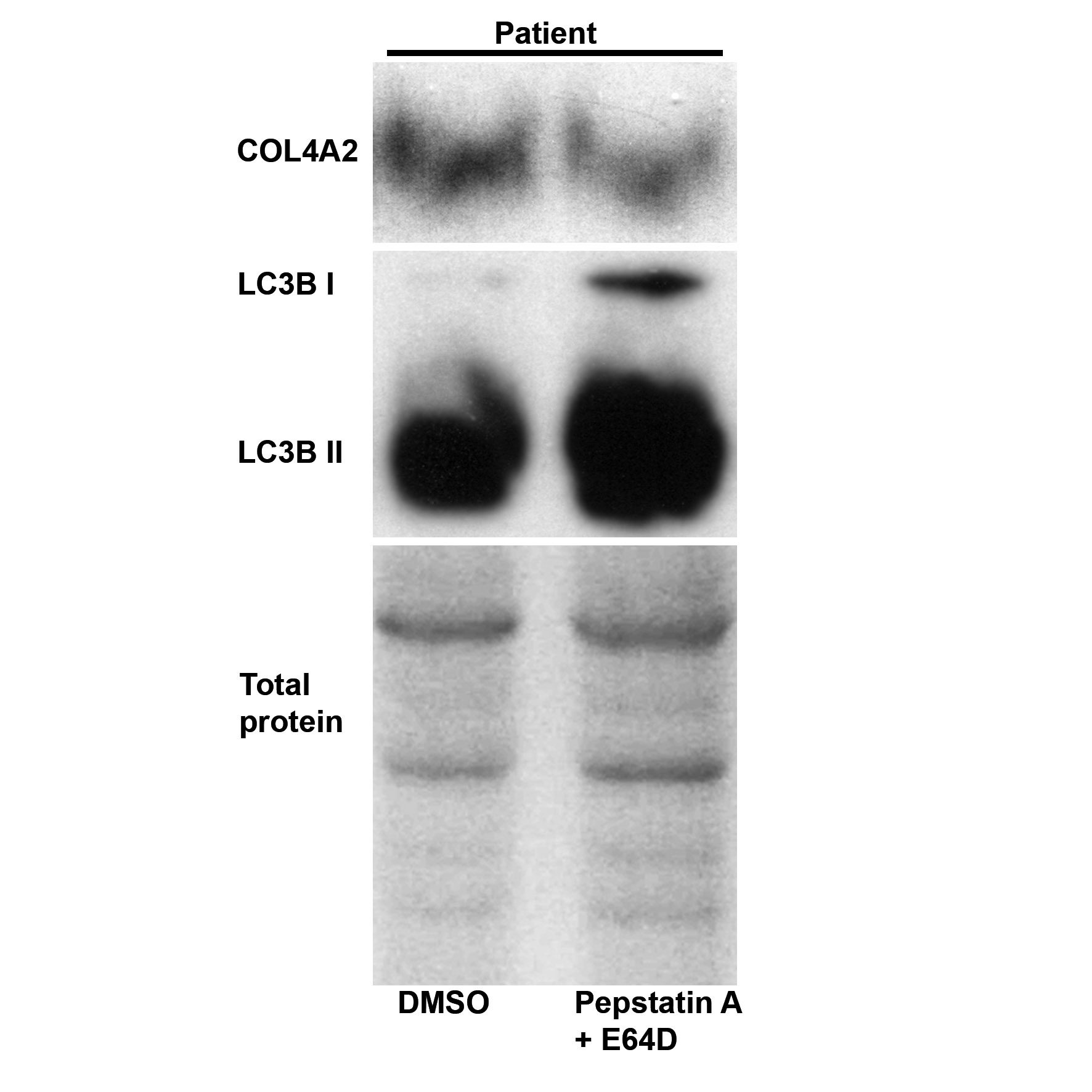
**

**Supplemental Figure 5:** Analysis of COL4A2 protein levels by Western blot in patient cells treated with the lysosome inhibitors pepstatin A and E64D. Western blotting against LC3B was used as a positive control for treatment as E64D/pepstatin A leads to increase in LC3B levels (1).


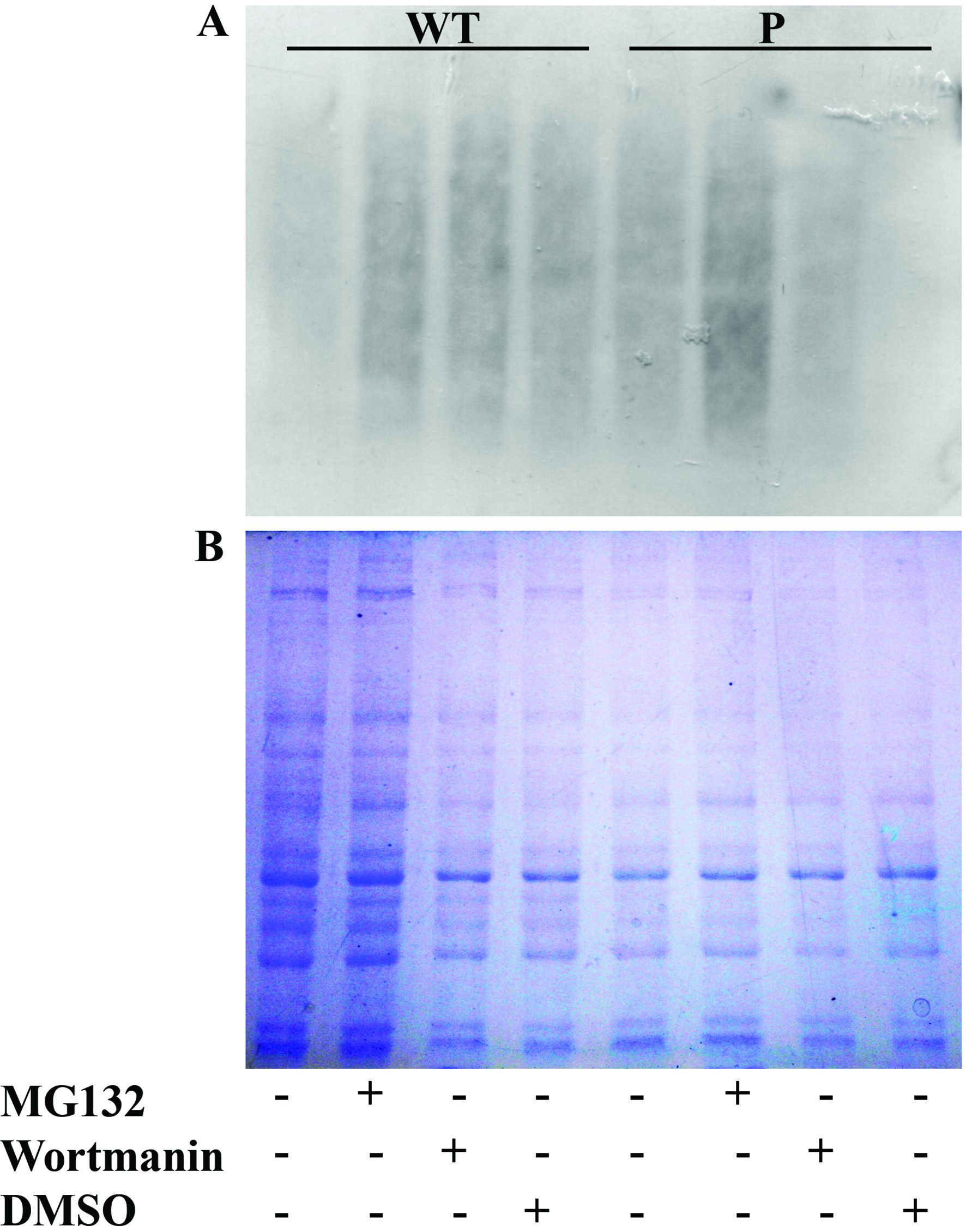


**Supplemental Figure 6:** (A) the full membrane from the ubiquitin western blot in Fig 7. (B) Entire coomassie stained gel used in Fig 7.


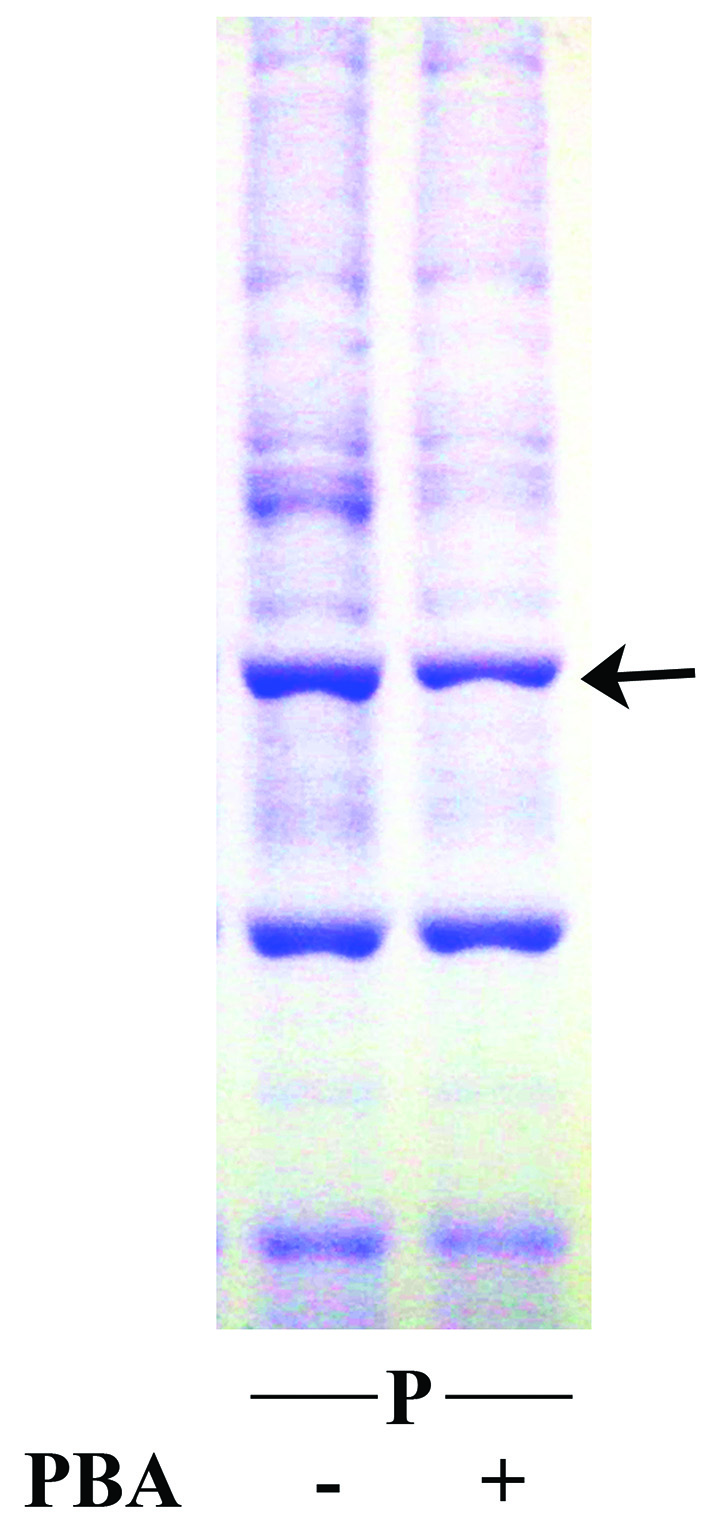


**Supplemental Figure 7:** Entire coomassie stained gel used in Fig 8. Protein band used is highlighted by black arrow.

**References**

1. [Tanida, I., Minematsu-Ikeguchi, N., Ueno, T. and Kominami, E.(2005) Lysosomal turnover, but not a cellular level, of endogenous LC3 is a marker for autophagy.](http://www.ncbi.nlm.nih.gov/pubmed?term=Tanida%20I%5BAuthor%5D&cauthor=true&cauthor_uid=16874052) *[Autophagy,](http://www.ncbi.nlm.nih.gov/pubmed?term=Tanida%20I%5BAuthor%5D&cauthor=true&cauthor_uid=16874052)* **[1](http://www.ncbi.nlm.nih.gov/pubmed?term=Tanida%20I%5BAuthor%5D&cauthor=true&cauthor_uid=16874052)**[, 84-91.](http://www.ncbi.nlm.nih.gov/pubmed?term=Tanida%20I%5BAuthor%5D&cauthor=true&cauthor_uid=16874052)
